# Corrosion Resistance of 3D Printed Ti6Al4V Gyroid Lattices with Varying Porosity

**DOI:** 10.3390/ma15144805

**Published:** 2022-07-09

**Authors:** Rachael Sharp, Matthew H. Pelletier, William R. Walsh, Cambre N. Kelly, Ken Gall

**Affiliations:** 1 Surgical and Orthopaedic Research Laboratories, Prince of Wales Clinical School, University of New South Wales, Sydney 2052, Australia; rachael.sharp@unsw.edu.au (R.S.); w.walsh@unsw.edu.au (W.R.W.); 2 Department of Mechanical Engineering and Materials Science, Duke University, Durham, NC 27708, USA; cambre.kelly@duke.edu (C.N.K.); kag70@duke.edu (K.G.)

**Keywords:** corrosion, gyroid, polarisation, tafel, Ti6Al4V

## Abstract

Corrosion of medical implants is a possible failure mode via induced local inflammatory effects, systemic deposition and corrosion related mechanical failure. Cyclic potentiodynamic polarisation (CPP) testing was utilized to evaluate the effect of increased porosity (60% and 80%) and decreased wall thickness in gyroid lattice structures on the electrochemical behaviour of LPBF Ti6Al4V structures. The use of CPP allowed for the landmarks of breakdown potential, resting potential and vertex potential to be analysed, as well as facilitating the construction of Tafel plots and qualitative Goldberg analysis. The results indicated that 60% gyroid samples were most susceptible to the onset of pitting corrosion when compared to 80% gyroid and solid samples. This was shown through decreased breakdown and vertex potentials and were found to correlate to increased lattice surface area to void volume ratio. Tafel plots indicated that despite the earlier onset of pitting corrosion, both gyroid test groups displayed lower rates of corrosion per year, indicating a lower severity of corrosion. This study highlighted inherent tradeoffs between lattice optimisation and corrosion behaviour with a potential parabolic link between void volume, surface area and corrosion being identified. This potential link is supported by 60% gyroid samples having the lowest breakdown potentials, but investigation into other porosity ranges is suggested to support the hypothesis. All 3D printed materials studied here showed breakdown potentials higher than ASTM F2129′s suggestion of 800 mV for evaluation within the physiological environment, indicating that under static conditions pitting and crevice corrosion should not initiate within the body.

## 1. Introduction

Titanium and titanium alloys are the most prevalent materials utilized within the orthopaedic space, with over 1000 tons of titanium devices being implanted worldwide every year [1]. High specific strength, good biocompatibility and good corrosion resistance when compared to other metals [2,3,4,5] make it a popular choice. However, despite this good corrosion resistance, all metals undergo a finite level of corrosion, particularly in the hostile environment of the human body, with the resulting corrosion remaining as a failure mode [6]. This generalized acceptance of titanium’s good corrosion resistance has led to studies investigating the effects of optimizing geometry and physical composition with little to no consideration of the impact on corrosion performance. These studies lack electrochemical endpoint testing, leaving an open question of the effect of design changes on the corrosion behaviour.

Potential adverse implications of corrosion include local inflammatory effects, systemic deposition, toxicity, and an overall reduction in mechanical stability with corrosion acting synergistically with wear to further accelerate the degradation [1,6,7,8,9,10]. These complications arise from the dissolution of the passive surface oxide layer leading to an increased release of metallic ions [11,12,13]. These complications have been observed in retrieval studies of Ti6Al4V implants, with reports of yellow nail syndrome, hypersensitivity, peri-implantitis, vanadium toxicity and a hypothesized link between aluminum ions and Alzheimer’s disease arising in research [1,14,15,16,17,18,19,20,21,22,23].

Currently, there is a rapid increase in both research and application of additively manufactured (AM) custom-made designs [24]. The clinical benefits of additive manufacturing in orthopedics are evidenced by reports of earlier mobilization rates and increased implant stability [25,26,27,28,29,30,31]. These clinical benefits have been researched and attributed to the creation of patient-specific implants with complex topologies and physical lattice structures to increase the implant porosity and decrease the effects of stress shielding [25,26,27,28,29,30,31]. Increasing void volume is a leading method utilized to optimize the osseointegration and increase load sharing abilities of an implant, with de Vasconellos et al. demonstrating greater percentages of new bone growth in 40% porous samples compared to 30% samples through histomorphometric studies [26]; multiple other studies have confirmed the ability for additively manufactured lattices to lower the elastic modulus of titanium from 100–110 GPa to that closer to cortical bone (10–30 GPa) [28,32,33]. These lattice structures aim to increase the porosity through the introduction of voids in either a random manner as seen in foams, or through a uniform repeated unit with one method being triply periodic minimal surfaces (TPMS). TPMS structures allow for both the elastic modulus and porosity to be tailored to mimic natural bone, whilst also providing a high level of performance predictability and reliability between samples [34].

One common TPMS structure being utilised in lattice-based implants is the gyroid. This is typically selected due to its desirable properties of having a high surface area to volume ratio, smooth transitions between unit cells, high toughness, and the ability to withstand elevated stresses [34,35,36]. Titanium gyroid lattices with porosities between 50% and 80% have elastic properties similar to that of trabecular and cortical bone, whilst also being able to withstand physiological loading conditions of everyday movement [34]. Recently, Kelly et al. demonstrated a non-linear relationship between osseointegration shear strength and titanium alloy gyroid implants with varied porosity in an ovine cortical model, concluding the porosity and thus surface area of the implant are important factors for stabilization. These findings are supported by experiments showing that the gyroid lattice is suited to biomorphic scaffold design in tissue engineering and has a superior ability to promote cell differentiation and proliferation when compared to other traditional arrangements [37,38]. Additionally, the corrosion of 80% gyroid lattices have recently been explored with Qin et al. demonstrating that Zn-0.7Li gyroids displayed lower weight loss than their bulk equivalent [39], whist another study showed that gyroid lattices exhibit higher polarisation resistance compared to 80% primitive and diamond lattices when exposed to a potassium hydroxide solution, suggesting gyroids have the strongest resistance to corrosion within the TPMS category [40].

At present, research into the electrochemical behaviour has investigated differences between additive manufactured solid samples and their wrought equivalent, identifying an increased risk of pitting onset produced in AM manufactured parts, and identifying that the electrochemical behaviour of wrought samples cannot be extrapolated to modern AM produced implants as once assumed [8,41,42]. By extension, lattice structures may have the potential to further increase this risk of pitting onset through the introduction of voids and crevices whilst also increasing the surface area exposed.

The purpose of the present study is to investigate the effect of introducing TPMS lattices with varying porosity on the electrochemical performance of 60% and 80% porous gyroid lattices created by laser powder bed fusion (LPBF), and solid control samples produced in the same manner. The 60% and 80% gyroid test groups that were selected as a study by Kelly et al. revealed that 60% samples experienced higher rates of osseointegration and increased mechanical fixation strength in a bicortical defect model, whilst samples exceeding 80% porosity were found to show diminishing bone ingrowth and strength [43]. Key identifiers from polarisation testing and qualitative analysis allowed for both the severity and susceptibility of samples to be compared, with an initial hypothesis being that an increase in porosity will increase both the susceptibility and severity of corrosion encountered.

## 2. Materials and Methods

### 2.1. Sample Preparation

Test samples featuring gyroid structures of 60% and 80% porosity as well as solid control samples were fabricated according to ASTM F3001 by laser powder bed fusion (LPBF) techniques (ProX DMP320, 3D Systems, Rock Hill, SC, USA) using Ti6Al4V ELI powder under an inert argon atmosphere. All samples’ external dimensions were 25 mm length by 10 mm width by 5 mm thickness, with the void volume of the gyroid structures altered through varying the wall thickness. Table 1 displays the theoretical surface area, porosity and volume of each test group as extrapolated from CAD (SolidWorks).

Excess powder was removed, and all samples underwent hot isostatic pressing (HIP) per ASTM F3001 (2 h, 900 C, 1000 bar) prior to removal from the build plate by wire electrical discharge machining. After removal from the build plate, samples underwent a surface blasting treatment and passivation in nitric acid prior to electrochemical analysis. 

The samples were imaged with stereozoom and scanning electron microscopes to survey the initial surface texture. Following this, surface roughness measurements were taken with a MarSurf PS10 mobile roughness instrument and were repeated 5 times at varying locations on the flat surface of the sample. The stylus was set to a traversing length of 3 mm and both the arithmetic mean surface deviation (Ra) and the 10-point mean surface deviation (Rz) were recorded. 

### 2.2. Cyclic Potentiodynamic Polarisation (CPP)

Cyclic potentiodynamic polarisation (CPP) testing was conducted on 6 samples from each test group as described by Munir et al. [44] and in accordance with ASTM Standard F2129-19 [45]. Test sample holders were constructed and insulated by three coatings of stop-off lacquer, to prevent corrosion of the stainless-steel clamp and crevice corrosion between the sample holder and the Ti6Al4V specimen.

Prior to every cyclic potentiodynamic test, the corrosion vessel (700 mL cylindrical flask) was cleaned by scrubbing and three rinses each of household detergent, de-ionised water, 95–100% ethanol and phosphate buffer saline (PBS) solution. CPP testing was completed with the use of a three-electrode polarisation cell featuring the Ti6Al4V sample (working electrode), an Ag/AgCL reference electrode (3 M KCl) and platinum mesh counter electrode submerged into the PBS solution regulated at a pH of 7.4 and temperature of 37 °C. The electrochemical cell was de-aerated throughout the test via nitrogen (N_2_) gas bubbling at a rate of 150 cm^3^/min. The open circuit potential was recorded after 60 min of de-aeration under a no current load. Following this, the forward scan was initiated with a speed of 1 mV/s and a current threshold of 1 A at which the scan was then reversed.

### 2.3. Post Corrosion Analysis

Post corrosion, the samples were removed from the potentiostat and photographed with corrosion by-products on the surface. Following this they were cleaned by submersion in dichloromethane, followed by overnight submersion in MMA polymer to remove lacquer stains or discoloration on the surface. Finally, high-pressure water was applied through a high-pressure washer, at pressures of 120 bar, to remove corrosion by-products from within the structure. During the high-pressure water cleaning the samples were clamped in place, with the polymeric vice clamp contacting the edges of the sample that did not undergo corrosion and were not under investigation. 

Quantitative data was collected through both cyclic potentiodynamic polarisation (CPP) curves and Tafel plots. CPP curves were exported from Nova software into MATLAB with a smoothing factor of 0.0005 applied to reduce the effect of noise. Multiplication factors of 0.753 and 0.854 were applied for 60% and 80% porous samples, respectively, with each multiplication factor representing the ratio of available surface area for solid samples to porous samples. These factors ensured all CPP landmark data were normalised to an exposed surface area of 351.6 mm^2^ to match the solid test group. Tafel plots were then constructed with OriginPro plotting software utilising 150 mV of data either side of the OCP or resting potential. Corrosion parameters were extrapolated from OriginPro, with the corrosion rate being calculated through Faraday’s equation as shown below. As all tests were conducted on Ti6Al4V alloy the density (*ρ*) was taken to be 4.5 g/cm^2^ and equivalent weight (EW) of 11.9 g [46,47]. As only half of each sample was available to corrode due to application of stop-off lacquer, the area (A) was taken to be half of the theoretical surface area shown in Table 1.

(1) $$\mathrm{Corrosion}\;\mathrm{Rate}=\frac{\left(3.272\times 10^{-3}\right)\times \frac{\left(J_{corr}\right)}{10^{-6}}\times \mathrm{EW}}{\rho A}\;\left(\frac{\mathrm{mm}}{\mathrm{year}}\right)$$

Qualitative analysis was completed through stereo-light microscopy and scanning electron microscopy at multiple magnification levels. The images obtained were then utilized to perform two methods of semi-quantitative analysis of the spread and severity. The spread was determined with MATLAB image analysis software to determine regions of discoloration and corrosion damage as a percentage of total area, whilst a modified Goldberg grading scale (as shown in Table 2) was utilised to determine the severity of corrosion encountered on the surface.

Statistical analyses for all quantitative and qualitative grading were performed by a one-way ANOVA using IBM SBSS statistics 26 software (IBM, Armonk, NY, USA) followed by post hoc comparisons between test groups. These were performed with a null hypothesis condition of *p* < 0.05 to determine statistical significance. A student *t*-test under the same null hypothesis condition was performed for the surface roughness as only data from solid and 60% test groups were obtained. Additionally, some conclusions were generated with *p* values close to or outside of this null hypothesis. These conclusions were correlated through multiple analysis methods to account for the large amounts of scatter as predicted in ASTM G16 Standard [48].

## 3. Results

### 3.1. Pre-Corrosion Analysis

**Figure 1:**
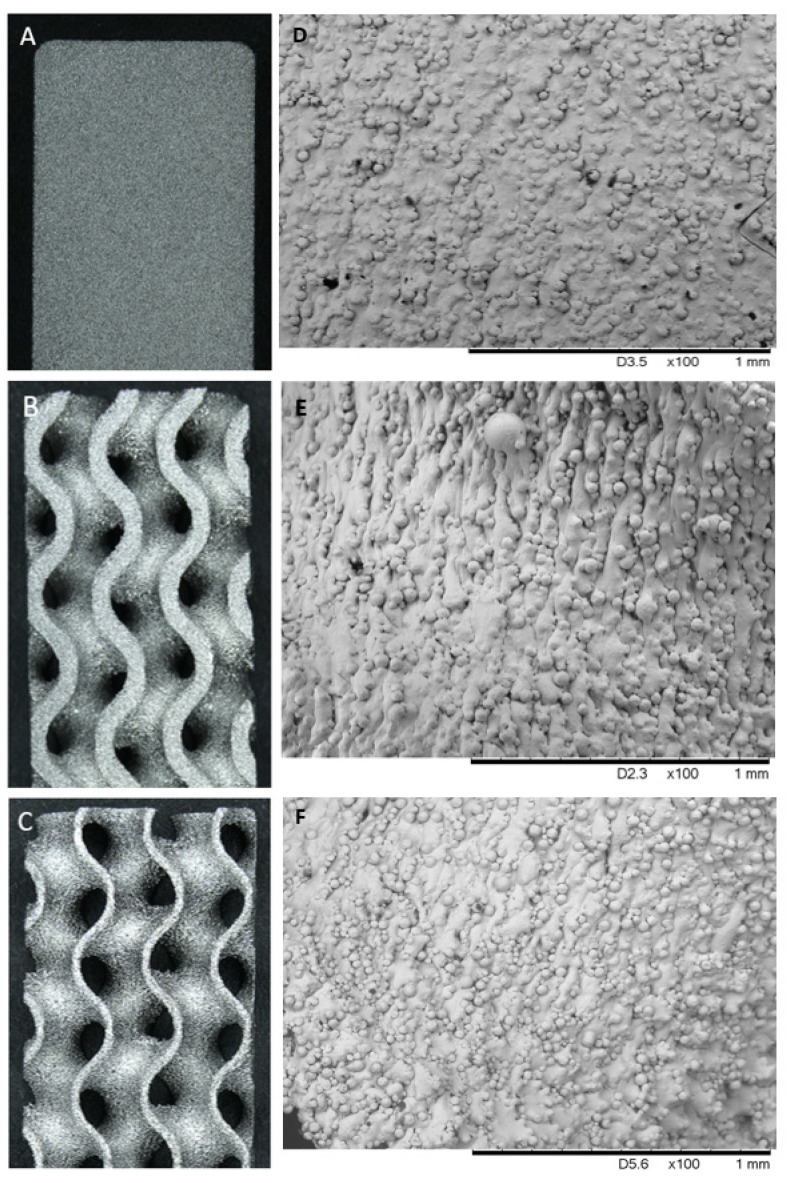
Stereozoom surface images of a LPBF Ti6Al4V produced; (**A**) solid sample; (**B**) 60% porous gyroid sample; (**C**) 80% porous gyroid sample. SEM surface images at 100× magnification of (**D**) solid sample; (**E**) 60% porous gyroid sample; (**F**) 80% porous gyroid sample. SEM images displayed increased definition and proportion of beads and “rippling” on the surface of both porous test groups.

Stereozoom and SEM images at 100× magnification showed all samples to have a rough surface with high proportions of sintered powder beads at the surface creating a functional porous structure, as shown in Figure 1. When comparing solid samples to both groups of porous samples under SEM, the gyroid lattice surfaces were shown to exhibit a “rippled” surface creating unidirectional folds. Additionally, both the 60% and 80% samples appeared to exhibit both a higher definition and proportion of sintered beads at the surface in comparison to the solid samples.

**Figure 2:**
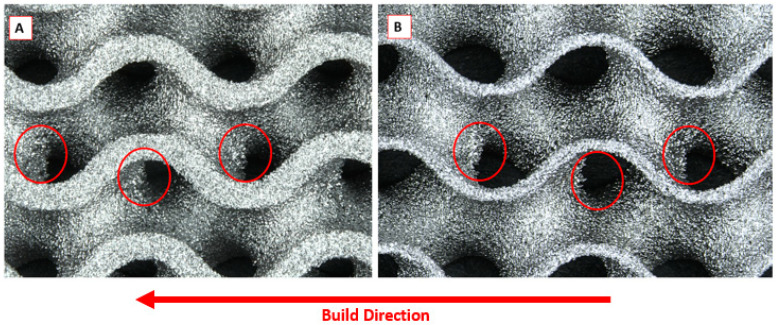
Stereozoom surface images of dross material (circled in red) observed in (**A**) 60% porous Ti6Al4V gyroid sample and (**B**) 80% porous Ti6Al4V gyroid sample produced by LPBF.

Dross material was observed on both the 60% and 80% samples indicating downward facing surfaces in relation to the build direction (Figure 2). The dross material was found to increase both the variability and absolute surface roughness when compared to the concave, convex and boundary proportions of the test samples.

Surface roughness measurements were then taken along the raised edges of both solid and 60% samples. These measurements utilized at 3 mm stylus traversing length, resulting in the 60% sample recordings being completed on the flat edge created at the boundary and no measurements being completed for 80% samples due to the lack of a straight edge. These results showed increased surface roughness for 60% samples with the R_a_ and R_z_ values being higher than solid samples as shown in Table 3.

### 3.2. Polarisation Results

**Figure 3:**
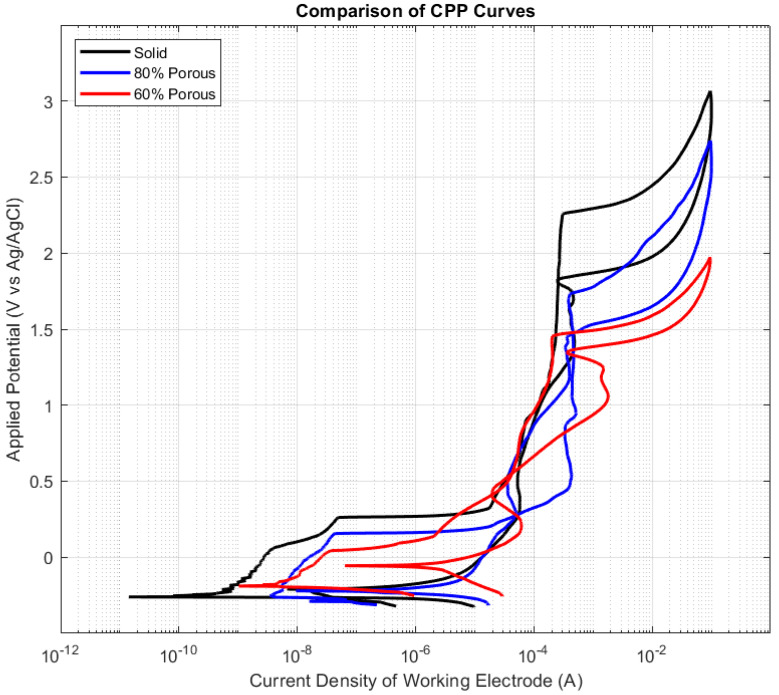
Comparison of example cyclic polarisation curves with solid samples shown in black; 80% porous gyroid sample shown in blue; 60% porous gyroid sample shown in red. All sample results were normalised to match the solid sample’s theoretical surface area of 351.6 mm^2^. Highest breakdown and vertex potentials observed in solid samples followed by 80% then 60%.

Through comparing the potentiodynamic polarisation curves after surface area adjustment, it was observed that solid samples displayed superior corrosion resistance as shown by higher breakdown and vertex potentials. This was followed by 80% porous samples and then 60% porous samples which were seen to have the most relative susceptibility to the onset of pitting corrosion, as shown in Figure 3.

It was observed that the majority of samples (12/18) did not feature an intersection between the reverse scan and forward scan as described in ASTM F2129 standards [45]. However, all samples featured a distinct change of gradient where the reverse scan approached the forward scan. This change of gradient was closely aligned with the behaviour of re-passivation (Figure 1A) rather than the open hysteresis loop that occurs in the absence of passivation (Figure 1B of ASTM F2129) [45]. This change of gradient in the reverse scan when in close proximity of the forward scan was coined the “near passivation” potential, at which samples are assumed to have undergone passivation, yet did not intersect due to current elevations at high voltages.

Landmarks from the polarisation curves were extrapolated and expressed in Table 4. The open circuit or resting potentials showed no significant difference at low potentials; however, there were distinct differences observed between test groups with all other CPP plot landmarks. The solid samples displayed higher breakdown and vertex potentials, followed by 80% and 60% indicating that solid samples were more resistant to the onset of pitting corrosion. When investigating the rate of corrosion, the hysteresis between the near passivation point and the breakdown potential showed no statistical significance between the test groups. This resulted in no conclusions regarding the rate or severity of corrosion being made from cyclic potentiodynamic polarisation data alone.

### 3.3. Tafel Plot Results

**Figure 4:**
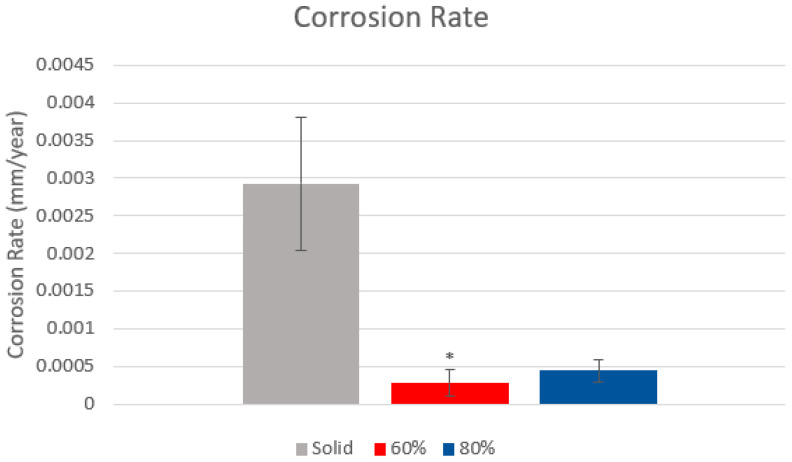
Average calculated Tafel corrosion rate of samples, with solid samples shown in grey, 80% porous gyroid in blue and 60% porous gyroid samples in red. * Indicates 60% samples having statistical significance to solid samples (*p* < 0.05) whilst 80% porous samples having *p* = 0.0532. (One-way ANOVA, Tukey).

Tafel plots were constructed and analysed for 4 out of 6 curves from each group utilizing data 150 mV either side of the OCP. Those that were not analysed were heavily affected by noise at low potentials and therefore presented multiple E_corr_ peaks; these were excluded from the analysis. Solid samples demonstrated a higher corrosion current density per area resulting in a higher corrosion rate per year, whilst both porous samples displayed similar results as summarized in Figure 4. These results indicated that porous samples underwent a lower rate or severity of corrosion than solid samples.

### 3.4. Post Corrosion Analysis

**Figure 5:**
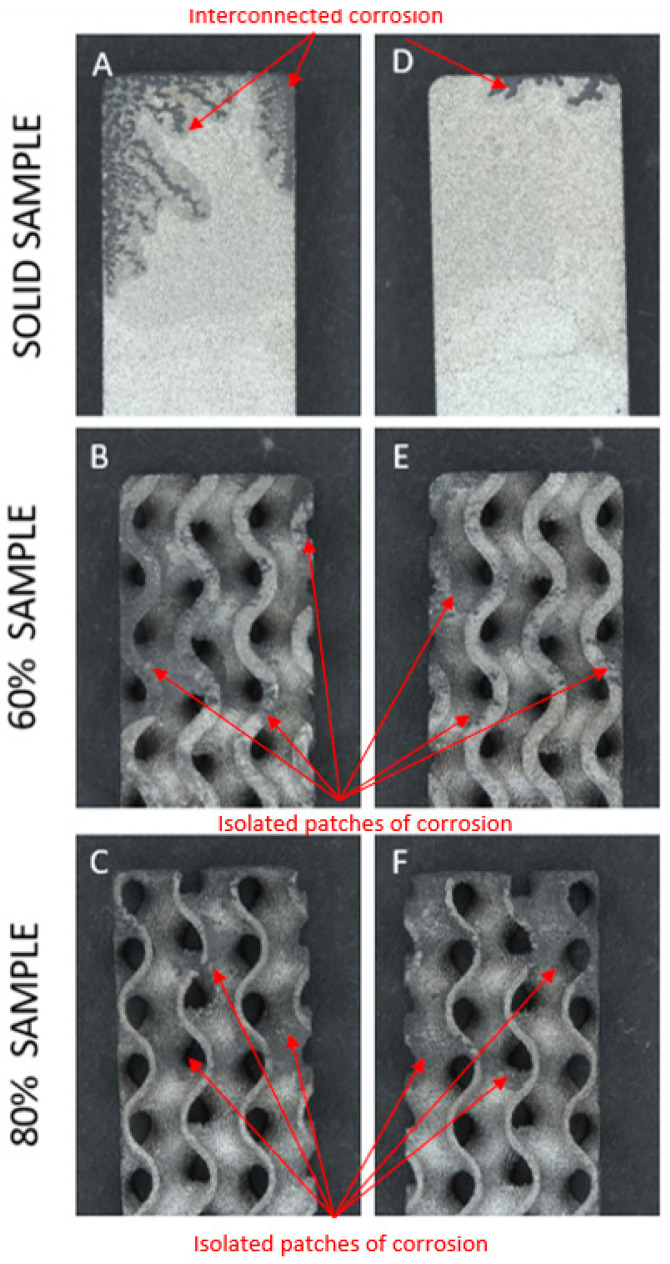
Stereozoom images of corrosion coverage on both faces of (**A**,**D**) solid sample; (**B**,**E**) 60% porous gyroid sample; and (**C**,**F**) 80% porous gyroid sample. All samples are Ti6Al4V and produced via LPBF. Corrosion was highly variable on sample surfaces and focused on raised edges and sample corners. Sections of corrosion indicated in red and characterized by discoloration and/or loss of material.

**Figure 6:**
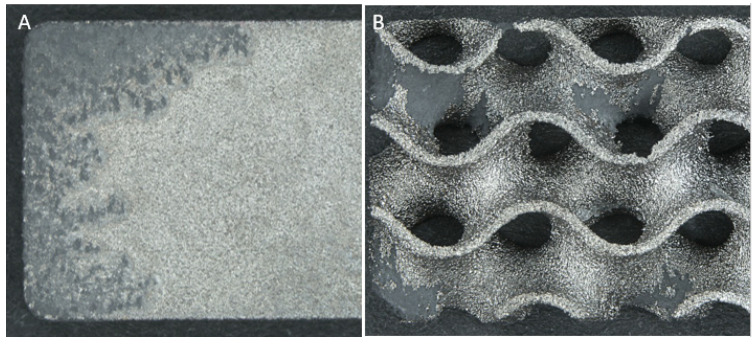
Stereozoom comparison of spread and interconnected finger-like propagations of corrosion on a (**A**) Ti6Al4V solid sample and isolated patches on a (**B**) 80% porous Ti6Al4V gyroid sample produced by LPBF.

Qualitative observations confirmed that all samples underwent corrosion, with all of them showing deposition of corrosion by-products on the surface when removed from the potentiostat. Following the cleaning process, five key observations were made as summarized below:

1. Within test groups there was a high amount of variability in the extent of corrosion observed;
2. Corrosion appeared to initiate and focus on the corners and raised edges of most samples (17/18) as expected. See Figure 5;
3. Corrosion of solid samples was highly interconnected, with finger-like propagations extending from the edges; whereas porous samples featured primarily distinct and isolated patches across the whole surface as shown in Figure 6;
4. The dross material observed in porous samples did not directly relate to an increase in corrosion, with many samples’ dross proportions being unaffected by corrosion;
5. All samples showed breakdown potentials higher than ASTM F2129′s suggestion of 800 mV for evaluation within the physiological environment.

Following qualitative observations, MATLAB image analysis was employed to quantify the spread of corrosion. As shown in Table 5 below, both porous samples showed greater spread of corrosion across the surface compared to solid samples, with no statistical difference seen between the 60 and 80% samples.

**Figure 7:**
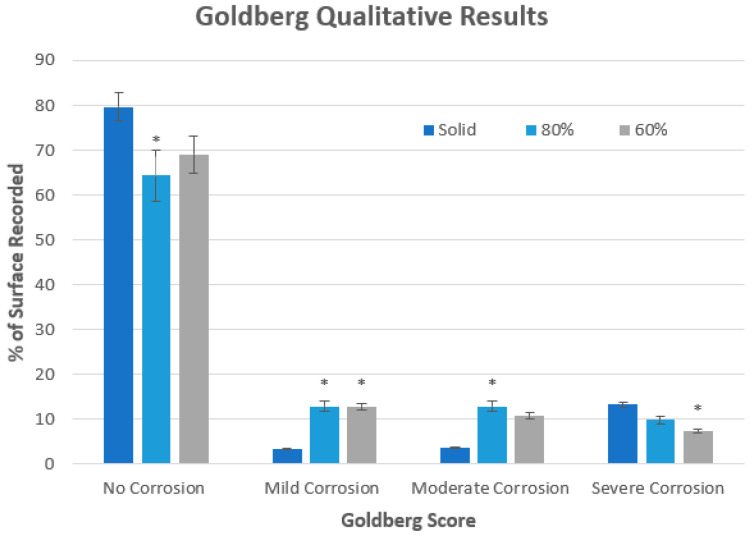
Semi-quantitative analysis of corrosion severity. Summary of no corrosion, mild, moderate, and severe corrosion on solid, 80% gyroid and 60% gyroid samples. * Indicates statistical significance to solid test group (*p* < 0.5). No significant differences were detected between the two porous groups (60% and 80% gyroids) (One-way ANOVA, Tukey).

The Goldberg scale revealed that solid samples experienced the highest percentages of both no corrosion and severe corrosion, whilst the porous samples showed a larger spread of mild and moderate corrosion, with a summary shown in Figure 7.

## 4. Discussion

**Figure 8:**
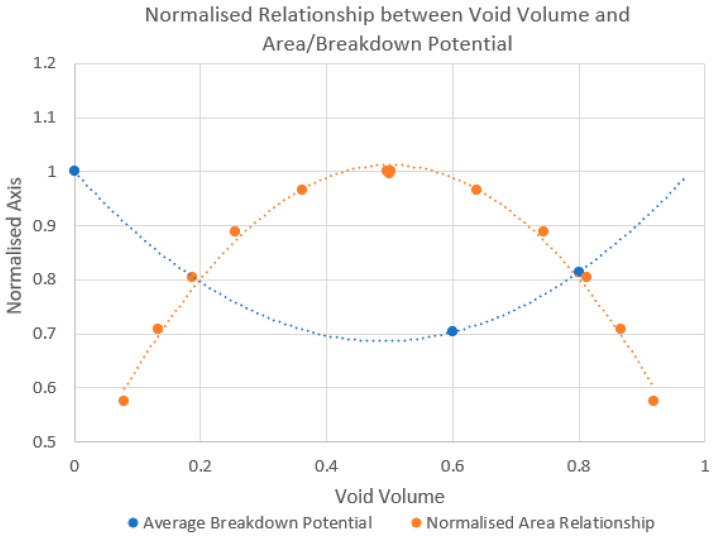
Generalized inverse parabolic relationship between breakdown potential and gyroid area to void volume ratio. Gyroid area curve adapted from Grobe-Braukman [49]. All values are normalised to a maximum value of one.

The potentiodynamic polarisation curves illustrated that 60% porous gyroid samples are slightly more susceptible to the onset of pitting corrosion, as indicated by the lower breakdown potentials when compared to 80% and solid samples. From this, no direct link was found between decreasing wall thickness and increasing pore size with 80% samples exhibiting higher breakdown and vertex potentials than 60% samples. These results instead are believed to follow a mathematical relationship between void volume and surface area as shown by Grobe-Brauckman [49]. This relationship follows a parabolic curve, and the breakdown potentials of each gyroid were found to show the inverse relationship as shown in Figure 8. The Grobe-Braukman curve implies that the largest surface area of a gyroid regardless of wall thickness or pore size will occur at 50% porosity, which is the location of zero mean curvature.

If applicable in this scenario, the relationship indicates that gyroid lattices of 50% porosity may be most susceptible to corrosion shown by having the lowest breakdown potentials, whilst high void volumes such as 95% may experience superior corrosion resistance similar to that of solid samples. As for other TPMS structures, the point of zero mean curvature with respect to the volume fraction may represent the most susceptible corrosion volume fraction of that lattice type. Future work would do well to include additional data points to further understand this relationship to overcome the inherent limitation of this study, which only investigated solid samples against 60% and 80% gyroid samples. In application for design of medical implants, the resistance to corrosion must be considered alongside mechanical performance and void volume for bone ingrowth. Thus, these factors often require complex tradeoffs depending on the intended clinical use of the device.

When evaluating the severity of corrosion, the opposite relationship to the susceptibility results was observed with solid samples experiencing the highest rates of corrosion as indicated by the increased hysteresis loop in CPP curves. This increased hysteresis loop represents the time taken for the sample to re-passivate after initial damage, with a larger voltage difference between the breakdown potential and the near-passivation or passivation point indicating an increased time to re-passivate and prevention of further corrosion. The severity was confirmed by Tafel plots where both porous groups exhibited lower average corrosion rates when compared to the solid test group. Despite 80% samples failing to reject the null hypothesis (*p* = 0.0532) through experiencing a large amount of scatter, the net average was lower than solid samples. This randomized scatter is identified within ASTMG16 [48] to be prevalent in electrochemical tests due to minor impurities in sample and/or test materials, and remains a challenge in electrochemical testing and statistical analysis. However, the combination of both qualitative, hysteresis potentials and qualitative analysis all correlate one another with the same observation that both porous groups exhibited a lower severity of corrosion when compared to the solid test group.

Qualitative analysis showed that the solid samples experienced higher rates of severe corrosion when compared to the 60% porous group (*p* < 0.05), and both porous groups instead exhibited higher proportions of mild and moderate corrosion. These observations were physically observed with solid samples showing interconnected and uniform corrosion propagating from the corners, whilst both porous groups were observed to result in isolated patches of varying severity of corrosion. These isolated patches may be caused by the increased rippled surface and the presence of unidirectional folds providing multiple regions for the forced initiation of crevice corrosion. These isolated patches were found not to correlate with the presence of dross material, instead focused at raised ridges and edges created at the external rectangular boundary. This observation may be related to the sharp change of build direction when manufactured, leading to the development of an uneven protective oxide film. These hypothesized theories support the earlier initiation of pitting and crevice corrosion in porous gyroid samples; however, gyroid lattices conversely showed a decreased severity of corrosion. The mechanisms behind this decreased severity are likely linked to the high and variable voltages, as explained in the limitations below. It is suggested that further experiments exploring the severity of corrosion of lattice structures be completed in future studies.

All samples showed breakdown potentials higher than ASTM F2129′s suggestion of 800 mV for evaluation within the physiological environment, indicating that under static conditions pitting and crevice corrosion should not initiate within the body [45]. This is supported as the solid samples in this study experienced the highest rate of corrosion at approximately 0.02 mm/year or 0.5 MPY which is comparable with existing Ti6Al4V implants, with gyroid samples showing a further decrease in rate [50,51].

## 5. Limitations

Cyclic potentiodynamic polarisation testing is the accepted method for assessing pitting corrosion for implant devices, and has been found to be a good general indicator for corrosion. CPP is limited to only investigate the occurrence of pitting and crevice corrosion on a stationary sample, meaning it does not account for movement at modular joints resulting in fretting corrosion, or the synergistic relationship between wear as well as excluding the effects of infection, inflammation and other biological responses that have been shown to affect the in vivo corrosion response [15,52,53].

Additionally, ASTM standards predict that oxygen evolution within PBS solution will occur around 0.5 V (SCE) [45]; at this point oxygen is released from the solution resulting in elevations of the measured current which are unrelated to the samples corrosion. For this reason, the current density was only investigated in Tafel plots of the forward scan where the potential applied had not yet reached 0.5 V at the point of measurement. These elevations at the threshold current are believed to have created the near passivation potential, resulting in the curves showing no intersection between forward and reverse scans.

High and variable potentials applied in this study. This test method was selected to ensure that breakdown of all materials occurred, as a constant current density was utilized rather than applied voltage. This method allowed for identification of breakdown potentials to accurately occur, however limited the accuracy of severity analysis as different samples underwent varying exposure times post breakdown potential with solid samples experiencing higher net voltages and increased loading compared to porous samples. These high potentials required an increased scan rate of 1 mV/s; these larger steps increased the effect of noise on the lower potentials leading to multiple Tafel plots exhibiting multiple peaks and therefore could not be used for Tafel corrosion rate analysis. To overcome this result, it is recommended to complete linear polarisation measurements between −500 mV and 100 mV with a scan rate of 0.167 mV/s prior to potentiodynamic polarisation to enable Tafel plot construction with decreased risk of low voltage noise.

## 6. Conclusions

Cyclic potentiodynamic polarisation testing demonstrated that 60% porous gyroid samples experienced the lowest resistance to corrosion and had an increased susceptibility to the onset of pitting. This behaviour was contrary to the hypothesis that increased porosity would increase the corrosion, with the results aligning with a parabolic relationship between volume, surface area and susceptibility to corrosion. Additionally, solid samples showed higher rates of severe corrosion on the surface. This result is believed to be impacted by both the re-passivation speed of the samples and through the higher voltages encountered by solid samples. These two key observations in corrosion resistance and severity are clinically significant as they identify variations in the electrochemical properties with the same Ti6Al4V material through alteration of the physical shape and structure, which has not yet been widely documented in literature.

## Figures and Tables

**Table 1:** Sample details derived from CAD (SolidWorks) for solid samples, 60% samples, and 80% samples with 25 mm by 10 mm by 5 mm external dimensions.

	Solid Sample	60% Sample	80% Sample
Test Group Size (*n*)	6	6	6
Porosity (%)	0	60.1	80.8
Surface Area (mm^2^)	703	933	824
Volume (mm^3^)	747	298	144

**Table 2:** Modified Goldberg severity scale utilised to qualitatively grade the surface corrosion [8,44].

Score	Severity	Criteria
0	No Corrosion	No visible corrosion
1	Mild Corrosion	Surface is discolored or dull
2	Moderate Corrosion	Surface is discolored, features shallow pitting and unclear corrosion boundaries
3	Severe Corrosion	Deep pitting and loss of surface material. Clear corrosion boundaries

**Table 3:** Surface roughness measurements of solid samples were compared to 60% porous gyroid samples. Five repeat measurements of each sample were taken on the flat exterior edge and averaged. The 80% porous gyroids were excluded due to the lack of an available flat section. A student *t*-test was completed to determine significance between groups (*p* < 0.05).

	Solid Samples	60% Samples	*p* Value
R_a_ (μm)	4.25 ± 0.26	5.46 ± 0.36	0.012
R_z_ (μm)	23.55 ± 0.86	30.50 ± 1.39	0.003

**Table 4:** Cyclic potentiodynamic polarisation landmark summary—mean values and standard deviation for each landmark. ** Denotes statistical significance to both groups (*p* < 0.05). * Denotes statistical significance to solid samples (*p* < 0.05) (one-way ANOVA, Tukey).

	Solid Samples	60% Samples	80% Samples
Resting Potential (E_r_/OCP) (mV)	−381 ± 110	−247 ± 45	−252 ± 129
Breakdown Potential (E_b_) (mV)	2262 ± 245 **	1590 ± 135 *	1842 ± 128 *
Near Passivation Potential (E_np_) (mV)	1839 ± 34 **	1375 ± 24 **	1548 ± 59 **
Vertex Potential (E_v_) (mV)	3061 ± 141 **	2137 ± 115 *	2387 ± 195 *
Hysteresis (E_b_—E_np_) (mV)	423 ± 220	215 ± 122	294 ± 131

**Table 5:** Corrosion spread analysis, percentage and surface area that experienced corrosion. ** Denotes statistical significance to solid samples *p* < 0.05 (One-way ANOVA, Tukey).

	Solid Sample	60% Sample	80% Sample
Exposed surface area (mm^2^)	351.57	466.66	411.92
Corroded percentage (%)	14.10	29.66 **	32.22 **
Corroded surface area (mm^2^)	49.57	138.40 **	132.72 **

## Data Availability

Available upon reasonable request.

## References

[1] Manivasagam D., Dhinasekaran D., Rajamanickam A. (2010). Biomedical Implants: Corrosion and its Prevention—A Review. Recent Pat. Corros. Sci..

[2] Minnath M.A. (2018). 7—Metals and alloys for biomedical applications. Fundamental Biomaterials: Metals.

[3] Katti K., Verma D.K., Katti (2008). Materials for joint replacement. Jt. Replace. Technol..

[4] Saini M., Singh Y., Arora P., Arora V., Jain K. (2015). Implant biomaterials: A comprehensive review. World J. Clin. Cases.

[5] Kamachimudali U., Sridhar T.M., Raj B. (2003). Corrosion of bio implants. Sadhana.

[6] Urban R.M., Gilbert J.L., Jacobs J.J. (2005). Corrosion of Modular Titanium Alloy Stems in Cementless Hip Replacement. ASTM Int..

[7] Jacobs J.J., Gilbert J.L., Urban R.M. (1998). Current Concepts Review—Corrosion of Metal Orthopaedic Implants. J. Bone Jt. Surg..

[8] Mah D., Pelletier M.H., Lovric V., Walsh W.R. (2019). Corrosion of 3D-Printed Orthopaedic Implant Materials. Ann. Biomed. Eng..

[9] Gilbert J.L., Buckley C.A., Jacobs J.J. (1993). In vivo corrosion of modular hip prosthesis components in mixed and similar metal combinations. The effect of crevice, stress, motion, and alloy coupling. J. Biomed. Mater. Res..

[10] Aksakal B., Yildirim Ö.S., Gul H. (2004). Metallurgical failure analysis of various implant materials used in orthopedic applications. J. Fail. Anal. Prev..

[11] Davda K., Lali F., Sampson B., Skinner J.A., Hart A.J. (2011). An analysis of metal ion levels in the joint fluid of symptomatic patients with metal-on-metal hip replacements. J. Bone Jt. Surg. Br. Vol..

[12] Bradberry S.M., Wilkinson J.M., Ferner R.E. (2014). Systemic toxicity related to metal hip prostheses. Clin. Toxicol..

[13] Antoniou J., Zukor D.J., Mwale F., Minarik W., Petit A., Huk O.L. (2008). Metal ion levels in the blood of patients after hip resurfacing: A comparison between twenty-eight and thirty-six-millimeter-head metal-on-metal prostheses. J. Bone Jt. Surg..

[14] Kim K.T., Eo M.Y., Nguyen T.T.H., Kim S.M. (2019). General review of titanium toxicity. Int. J. Implant. Dent..

[15] Eliaz N.J.M. (2019). Corrosion of Metallic Biomaterials: A Review. Materials.

[16] Pina V.G., Dalmau A., Devesa F., Amigó V., Muñoz A.I. (2015). Tribocorrosion behavior of beta titanium biomedical alloys in phosphate buffer saline solution. J. Mech. Behav. Biomed. Mater..

[17] Engh C.A., Moore K.D., Vinh T.N., Engh G.A. (1997). Titanium Prosthetic Wear Debris in Remote Bone Marrow. A Report of Two Cases*. J. Bone Jt. Surg..

[18] Barbieri M., Mencio F., Papi P., Rosella D., Di Carlo S., Valente T., Pompa G. (2017). Corrosion behavior of dental implants immersed into human saliva: Preliminary results of an in vitro study. Eur. Rev. Med. Pharmacol. Sci.

[19] Furrer S., Hofmeier K.S., Grize L., Bircher A.J. (2018). Metal hypersensitivity in patients with orthopaedic implant complication. A retrospective clinical study. Contact Dermat..

[20] Haynes D.R., Rogers S.D., Hay S.J., Pearcy M.J., Howie D.W. (1993). The differences in toxicity and release of bone-resorbing mediators induced by titanium and cobalt-chromium-alloy wear particles. J. Bone Jt. Surg..

[21] Rogers S.D., Howie D.W., Graves S.E., Pearcy M.J., Haynes D.R. (1997). In Vitro Human Monocyte Response to Wear Particles of Titanium Alloy Containing Vanadium or Niobium. J. Bone Jt. Surg. Br. Vol..

[22] Safioti L., Kotsakis G.A., Pozhitkov A.E., Chung W.O., Daubert D.M. (2017). Increased Levels of Dissolved Titanium Are Associated With Peri-Implantitis—A Cross-Sectional Study. J. Periodontol..

[23] Berglund F., Carlmark B. (2011). Titanium, Sinusitis, and the Yellow Nail Syndrome. Biol. Trace Elem. Res..

[24] Ford S.L.N. (2014). Additive Manufacturing Technology: Potential Implications for U.S. Manufacturing Competitiveness. J. Int. Commer. Econ..

[25] Siu T.L.T., Rogers J.M., Lin K., Thompson R.G., Owbridge M. (2018). Custom-Made Titanium 3-Dimensional Printed Interbody Cages for Treatment of Osteoporotic Fracture-Related Spinal Deformity. World Neurosurg..

[26] Vasconcellos L.M.R., Leite D.d.O., Oliveira F.N.d., Carvalho Y.R., Cairo C.A.A. (2010). Evaluation of bone ingrowth into porous titanium implant: Histomorphometric analysis in rabbits. Braz. Oral Res..

[27] Tsai P.I., Wu M.H., Li Y.Y., Lin T.H., Tsai J., Huang H.I., Lai H.J., Lee M.H., Chen C.Y. (2021). Additive-manufactured Ti-6Al-4 V/Polyetheretherketone composite porous cage for Interbody fusion: Bone growth and biocompatibility evaluation in a porcine model. BMC Musculoskelet. Disord..

[28] Park P.J., Lehman R.A. (2020). Optimizing the Spinal Interbody Implant: Current Advances in Material Modification and Surface Treatment Technologies. Curr. Rev. Musculoskelet. Med..

[29] Zhang L.C., Chen L. (2019). A Review on Biomedical Titanium Alloys: Recent Progress and Prospect. Adv. Eng. Mater..

[30] Singh S., Ramakrishna S. (2017). Biomedical applications of additive manufacturing: Present and future. Curr. Opin. Biomed. Eng..

[31] Prasad K., Bazaka O., Chua M., Rochford M., Fedrick L., Spoor J., Symes R., Tieppo M., Collins C., Cao A. (2017). Metallic Biomaterials: Current Challenges and Opportunities. Materials.

[32] Maietta S., Gloria A., Improta G., Richetta M., de Santis R., Martorelli M. (2019). A Further Analysis on Ti6Al4V Lattice Structures Manufactured by Selective Laser Melting. J. Healthc. Eng..

[33] Wu S.-h., Li Y., Zhang Y.-Q., Li X.-K., Yuan C.-F., Hao Y.-L., Zhang Z.-Y., Guo Z. (2013). Porous titanium-6 aluminum-4 vanadium cage has better osseointegration and less micromotion than a poly-ether-ether-ketone cage in sheep vertebral fusion. Artif. Organs.

[34] Kelly C.N., Francovich J., Julmi S., Safranski D., Guldberg R.E., Maier H.J., Gall K. (2019). Fatigue behavior of As-built selective laser melted titanium scaffolds with sheet-based gyroid microarchitecture for bone tissue engineering. Acta Biomater..

[35] Al-Ketan O., Rowshan R., Abu Al-Rub R.K. (2018). Topology-mechanical property relationship of 3D printed strut, skeletal, and sheet based periodic metallic cellular materials. Addit. Manuf..

[36] Al-Ketan O., Lee D.W., Rowshan R., Abu Al-Rub R.K. (2020). Functionally graded and multi-morphology sheet TPMS lattices: Design, manufacturing, and mechanical properties. J. Mech. Behav. Biomed. Mater..

[37] Timercan A., Sheremetyev V., Brailovski V. (2021). Mechanical properties and fluid permeability of gyroid and diamond lattice structures for intervertebral devices: Functional requirements and comparative analysis. Sci. Technol. Adv. Mater..

[38] Zhao D., Liang H., Han C., Li J., Liu J., Zhou K., Yang C., Wei Q. (2021). 3D printing of a titanium-tantalum Gyroid scaffold with superb elastic admissible strain, bioactivity and in-situ bone regeneration capability. Addit. Manuf..

[39] Qin Y., Yang H., Liu A., Dai J., Wen P., Zheng Y., Tian Y., Li S., Wang X. (2022). Processing optimization, mechanical properties, corrosion behavior and cytocompatibility of additively manufactured Zn-0.7 Li biodegradable metals. Acta Biomater..

[40] Łosiewicz B., Maszybrocka J., Kubisztal J., Skrabalak G., Stwora A. (2021). Corrosion Resistance of the CpTi G2 Cellular Lattice with TPMS Architecture for Gas Diffusion Electrodes. Materials.

[41] Abdeen D.H., Palmer B.R. (2016). Corrosion evaluation of Ti-6Al-4V parts produced with electron beam melting machine. Rapid Prototyp. J..

[42] Wang G., Wan Y., Wang T., Liu Z. (2017). Corrosion Behavior of Titanium Implant with different Surface Morphologies. Procedia Manuf..

[43] Kelly C.N., Wang T., Crowley J., Wills D., Pelletier M.H., Westrick E.R., Adams S.B., Gall K., Walsh W.R. (2021). High-strength, porous additively manufactured implants with optimized mechanical osseointegration. Biomaterials.

[44] Munir S., Pelletier M.H., Walsh W.R. (2016). Potentiodynamic Corrosion Testing. J. Vis. Exp. JoVE.

[45] (2019). Standard Test Method for Conducting Cyclic Potentiodynamic Polarization Measurements to Determine the Corrosion Susceptibility of Small Implant Devices.

[46] Gunawarman, Giatmana D.D., Ilhamdi, Affi J., Fonna S., Niinomi M., Nakai M. (2018). Corrosion resistance of new beta type titanium alloy, Ti-29Nb-13Ta-4.6Zr in artificial saliva solution. IOP Conf. Ser. Mater. Sci. Eng..

[47] (2006). Standard Practice for Calculation of Corrosion Rates and Related Information from Electrochemical Measurements.

[48] (2019). Standard Guide for Applying Statistics to Analysis of Corrosion Data.

[49] Große-Brauckmann K. (1997). Gyroids of Constant Mean Curvature. Exp. Math..

[50] Vijayaraghavan V., Sabane A.V., Tejas K. (2012). Hypersensitivity to Titanium: A Less Explored Area of Research. J. Indian Prosthodont. Soc..

[51] Almanza E., Pérez M.J., Rodríguez N.A., Murr L.E. (2017). Corrosion resistance of Ti-6Al-4V and ASTM F75 alloys processed by electron beam melting. J. Mater. Res. Technol..

[52] Souza J.C.M., Apaza-Bedoya K., Benfatti C.A.M., Silva F.S., Henriques B. (2020). A Comprehensive Review on the Corrosion Pathways of Titanium Dental Implants and Their Biological Adverse Effects. Metals.

[53] Dini C., Costa R.C., Sukotjo C., Takoudis C.G., Mathew M.T., Barão V.A.R. (2020). Progression of Bio-Tribocorrosion in Implant Dentistry. Front. Mech. Eng..

